# Minor effect of wind exposure and littoral slope on macrophyte characteristics in non-impacted lowland lakes of Poland

**DOI:** 10.3389/fpls.2023.1307453

**Published:** 2024-01-09

**Authors:** Agnieszka Kolada, Sebastian Kutyła, Aleksandra Bielczyńska

**Affiliations:** ^1^ Department of Freshwater Protection, Institute of Environmental Protection—National Research Institute, Warsaw, Poland; ^2^ Independent Scholar, Warsaw, Poland

**Keywords:** macrophytes, wind exposure, littoral slope, lakes, ecological status

## Abstract

Aquatic vegetation is a reliable indicator of the ecological condition of surface waters. Abundance, composition and spatial structure of aquatic communities are shaped by an array of factors, which include both natural abiotic features of an ecosystem and external influences. We investigated whether the physical features, i.e., wind exposure and slope of the lake basin, have a significant impact on the taxonomic composition and spatial structure of macrophyte communities from non-impacted, highly alkaline, lowland lakes of the European plains (Poland). We further examined whether these features can affect the classification of the ecological status of lakes assessed in accordance with the Water Framework Directive requirements. Morphological, botanical and physicochemical data from 260 transects in 16 non-disturbed lakes of Polish lowlands surveyed in the years 2011–2016 were analysed. For each transect, littoral slope and wind exposure were calculated. Additionally, the total phosphorus concentration was used as a proxy of water trophy. The relationships between environmental variables and macrophyte indices as well as the syntaxonomic composition of aquatic and rush vegetation (dependent variables) were analysed using multidimensional ordination techniques (redundancy analysis, variation partitioning and indicator values), correlation and regression analysis. Among the three analysed environmental factors (littoral slope, wind exposure and water trophy), in almost all cases the latter explained the highest variance in the macrophyte community, while the contribution of the first two was at most moderate, weak or usually statistically insignificant. However, lakes with steeper slopes were more frequently inhabited by stoneworts and had better ecological status than those with a gentle littoral shape. This may be attributed to the links between lake morphometry and rate of eutrophication, with deep lakes supporting more effective dilution of substances. Furthermore, lower light requirements of charophytes than of higher plants and the capacity to growth in unstable sediments facilitate charophyte establishment in deeper and steeper parts of the littoral over higher plants. Our findings suggest that in lowland lakes with relatively small areas, moderate depths and low wind exposure typical of European plains, slopes and weaving do not hamper vegetation development and do not negatively affect the macrophyte assessment of ecological status. In such ecosystems, eutrophication seems to be a more important factor determining aquatic vegetation than physical features.

## 1 Introduction

The composition and abundance of aquatic biological assemblages are shaped by an array of factors that determine the structure and functioning of their environments. The constantly increasing anthropogenic pressures on aquatic ecosystems has stimulated a multitude of studies on the effects of environmental gradients on aquatic biota. In Europe, these studies were largely intensified by the Water Framework Directive (EC, 2000), which regulates the assumptions of the European Union’s water policy. The directive provides rules that aim to halt deterioration of the EU waters and achieve good status for Europe’s rivers, lakes and groundwater. This piece of legislation introduced biological communities as the basis for assessing the quality of aquatic ecosystems, i.e., ecological status. The ecological status of waters reflects the deviation of the current state of the environment (in terms of biological, physicochemical and hydromorphological quality elements) from the reference state expected in undisturbed conditions. This approach, hence, strongly relates water status to external pressures affecting ecosystems.

Aquatic vegetation is one of the valuable biological quality indicators, as it clearly responds to an array of anthropogenic disturbances, such as eutrophication (Toivonen and Huttunen, 1995; Lyche Solheim et al., 2013), salinisation (Feld et al., 2023; Velthuis et al., 2023), acidification (Farmer, 1990; Muniz, 1990; Lacoul et al., 2011) or hydromorphological modifications (Mjelde et al., 2013; Kutyła et al., 2021). However, physical features of the environment, such as morphometric conditions (including wave exposure and shape of the littoral zone) are fundamental determinants of aquatic vegetation and may modify macrophyte response to external influences.

Wave activity is one of the key physical drivers of littoral habitats. Water movement affects shoreline erosion rates, sediment sorting and resuspension and, consequently, the distribution and composition of aquatic organisms. Offshore, wind exposure influences currents, thermal stratification, upwelling and lake turnover events (Mason et al., 2018). It increases sediment resuspension, hence water turbidity, and mechanically limits the occurrence of plants. In habitats exposed to strong waves, the substrate is usually sandy, while moderate and weak water movement supports increased sedimentation and results in a more muddy and nutrient-rich substrate. Thus, lowest wave activity promotes intensive development of flora, which constitute a shelter and food base for invertebrate fauna and fish (Keddy, 1982; Tolonen et al., 2001). Fetch and exposure of the nearshore to wind and waves have been found to be important factors in structuring ecosystems and different groups of biological communities (Randall et al., 1996; Uzarski et al., 2004; Cooper et al., 2014). For example, shore exposure controls corresponding types of nearshore vegetation structure, which in turn influence the diversity, structure and productivity of fauna including invertebrates, birds, reptiles and fish. Consecutively, the shape of the bottom and undulations in the littoral zone may affect the entire food web of the lake.

The clear effects of the shape of a lake littoral zone on habitat development and conditions were also reported (Håkanson, 1977; Duarte and Kalff, 1986; Jupp and Spence, 1997). In parts of the littoral with gentle slopes, the sedimentation is promoted, and the bottom substrate is usually of considerable thickness and rich in nutrients. In sites with steep slopes, where sedimentation is limited, the sediment thickness is usually low and the substrate is poor in nutrients. van Nes et al. (2002) demonstrated an effect of the littoral slope on light availability and, consequently, on the distribution and biomass of macrophytes. They found significant differences in macrophyte characteristics at slopes below and above 2%. Duarte and Kalff (1986) showed that with a slope of more than 2.24%, the biomass of submerged vegetation abruptly decreased, and this relationship was exponential. In 25 glacial lakes in North America analysed by Duarte and Kalff (1990), at littoral slopes exceeding 14.8%, no vegetation was found.

Physical parameters such as depth, littoral slope, wave activity and sediment types were demonstrated to be important determinants of the spatial structure and composition of lake vegetation (Duarte and Kalff, 1990; Blindow, 1992; Middleboe and Markager, 1997; Azzella et al., 2014). However, those studies made little effort to distinguish the state of biota due to natural processes (spontaneous succession) from those resulting from human activity modifying natural processes (anthropogenic degradation). Such changes may mask or expose the results of assessment of anthropogenic pressures, e.g., eutrophication or hydromorphological alterations and must be acknowledged in bioassessment systems.

In this study, we explored whether and how the physical features of a lake, wind exposure and littoral slope affect macrophyte community composition and spatial structure. We hypothesised that parts of the littoral zone with steep slopes and strong wave activity would host conditions unfavourable for dense macrophyte development and growth. This may negatively affect bioassessment results irrespective of the human pressure. We consider such downgrading unfounded, as ecological status should reflect the effects of human disturbances and not influences of intrinsic abiotic factors. Therefore, to test our hypothesis we selected lakes not exposed to anthropogenic pressure (in near-natural conditions) and examined how two physical factors, wind exposure and littoral slope, influenced vegetation characteristics and, thus, macrophyte-based classification of lake ecological status as defined by the WFD.

## 2 Materials and methods

### 2.1 Lake selection

**Figure 1:**
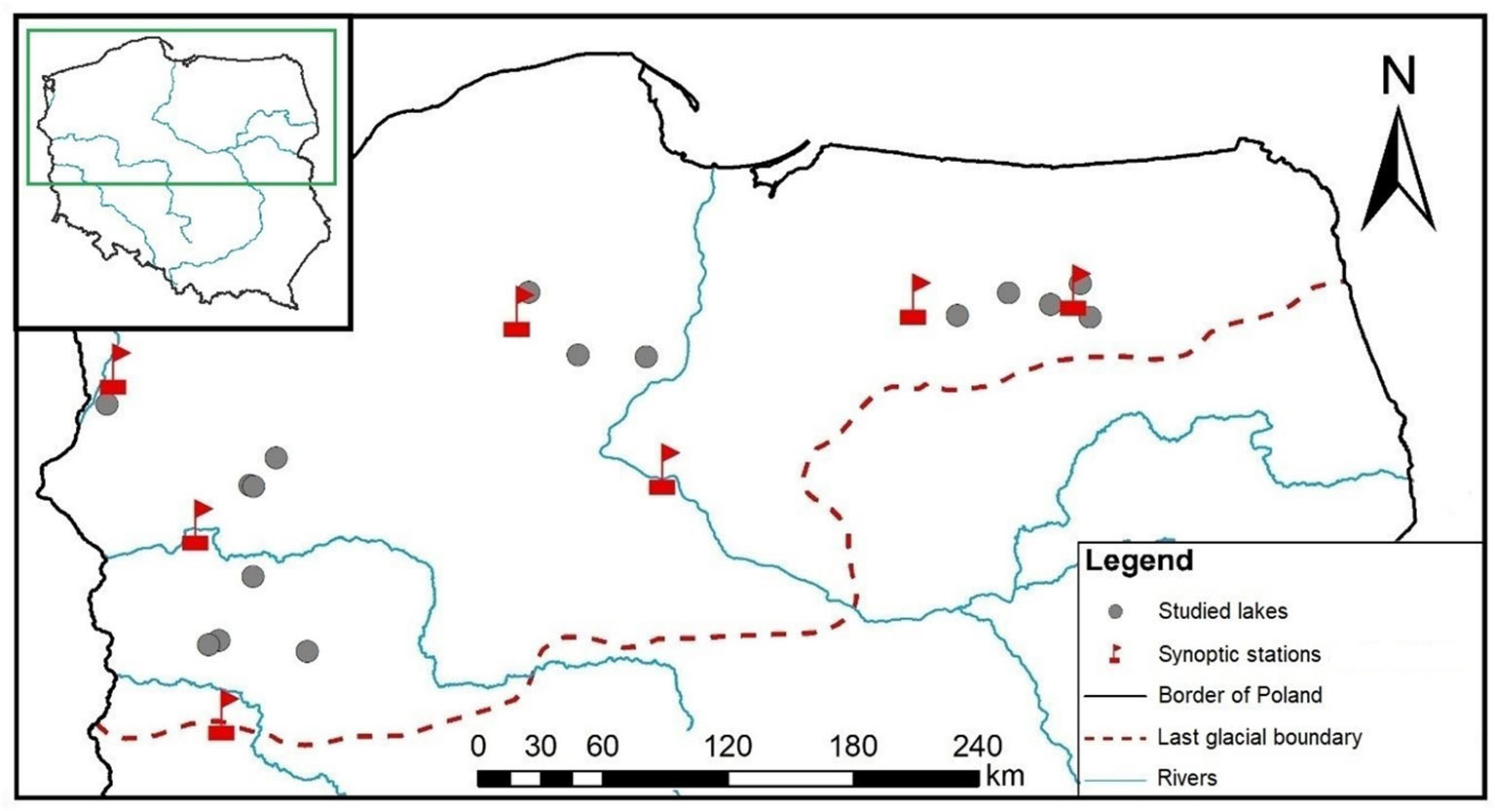
Location of the lakes and synoptic stations, from where the meteorological data were obtained.

We used the national monitoring database from the years 2011–2016 to derive the botanical, physicochemical and morphometric data analysed in this study. The database contains 520 lakes representative of Polish lowlands, i.e., lakes with high alkalinity (>1.2 meq l^-1^), low to moderate conductivity (<1000 µS cm^-1^) and non-coloured waters. To reduce the variation found in macrophyte responses due to anthropogenic pressures, we screened the database for lakes with no evidence of human-induced deterioration. We assumed that non-impacted lakes would be more suitable for testing our hypothesis, as in lakes affected by nutrient enrichment, the effects of physical lake features on macrophytes may be masked by eutrophication-induced modifications. The criteria for the selection of non-disturbed (reference) lakes can be found in Soszka et al. (2008). We identified 37 such lakes, of which for 16 lakes there were complete morphometric (see sec. 2.3) and adequate meteorological data (see sec. 2.4). These 16 lakes were selected for further analysis. They are evenly distributed across Polish lowlands (**Figure 1**), represent different geographical regions and vary in morphometric conditions, including a range of sizes and depths representative to the population of Polish lakes monitored under WFD (Kolada et al., 2005). Although their physicochemical and biological characteristics indicate non-disturbed conditions, their trophic gradients range between 0.015 and 0.049 mg l^-1^ of the total phosphorus (TP) concentration (**Supplementary Table 1**).

### 2.2 Macrophyte data

Macrophyte field surveys employed the transect method with 30 m wide transects set perpendicular to the shoreline and extending from the eulittoral zone to the maximum colonisation depth. According to the state monitoring method, the number of transects in a lake depends on the lake area and shoreline length (Ciecierska and Kolada, 2014), and for the analysed lakes they ranged from 8 to 27. Overall, data from 260 transects were analysed.

Macrophyte monitoring in Poland employs the synecological approach (Braun-Blanquet, 1964), where plant communities are considered the basic units, compatible with species in taxonomy. Plant community (association) is defined as an abstract vegetation unit that has a definite floristic composition and uniform physiology, and occurs in uniform habitat conditions. The term ‘community’ (syntaxon) is, thus, used for homogenous and uniform vegetation stands (Westhoff and van der Maarel, 1973, after Jensen, 1977), named after the dominating species. Plant units are recorded as long as they create stands with an area of at least 1 m^2^. The survey covered all groups of macrophytes, including hydrophytes (mosses, charids, potamids, nympheids and pleustophytes) and helophytes (emergent vegetation).

Of 155 plant communities recorded in the entire monitoring database, 69 were found in the 260 analysed transects. Of these, 46 were hydrophyte and 23 were helophyte communities (**Supplementary Table 3**). For each transect, data on the total macrophyte coverage (%COV), maximum colonisation depth (C_max_) and the relative % cover of each community in the total vegetated area within a transect, were collected.

Furthermore, the Ecological State Macrophyte Index (ESMI; Ciecierska and Kolada, 2014) was analysed. The multimetric ESMI includes the syntaxonomic composition component (Pielou’s index of evenness J’) and the abundance component (the colonisation index Z) as shown below:


ESMI=1−exp[−H'Hmax'×Niso.2.5×exp NP]

where: H’/H_max_ = J’ – Pielou’s index of evenness (Pielou, 1975); N/iso.2.5 = Z – colonisation index; N – total area vegetated; P – lake area [ha].

In the national assessment system, ESMI is calculated as one value for a lake based on the data compiled from all transects. In this study, the ESMI index was modified to enable index calculation for each transect separately (Kolada, 2016). In the modified formula (ESMI_TR_), the maximum colonisation depth on a transect (C_max_TR) was used as a surrogate of the total vegetated area in a lake (N), while the maximum depth of a lake (Z_max_) was used instead of its total area (P), as shown below:


ESMITR=1−exp[−J'×CmaxTR2,5×expCmaxTRZmax]

The influence of physical factors on the macrophyte condition was analysed for abundance and spatial structure indicators (**Supplementary Table 2**) and for macrophyte syntaxonomic composition (**Supplementary Table 3**).

### 2.3 Littoral slope calculation

For each transect, the individual slope was determined based on a raster map of the lake’s depth with a spatial resolution of 1 m x 1 m, prepared according to method presented by Urbański and Kryla-Straszewska (2010), using the ArcGIS for Desktop v. 10.1 (ESRI, 2012). First, the hardcopy isobathic plans of the lakes (data of the Inland Fisheries Institute from the years 1952-1968) were given a spatial reference using the *‘Georeferencing’* tool and the izobath screen digitization was performed. Next, the isolines were integrated into the current contour of the lake, derived from the Hydrographic Map of Poland in a 1:10,000 scale (Barszczyńska et al., 2013) updated by Grela et al. (2017), using the flexible fitting algorithm. Interpolation was performed using the *‘Topo to Raster’* method, which interpolates a hydrologically correct raster surface based on vector data, i.e., points, lines and polygons. It is based on the ANUDEM program developed by Hutchinson (1988; 1989). A map of an entire lake basin slope was generated from the raster map of the lake’s depth using the ‘*Slope’* tool (Burrough and McDonell, 1998).

The slopes for individual transects (linear objects) were calculated using the *‘Zonal Statistics as Table’* option. Transect length was determined based on macrophyte maximum colonisation depth (C_max_) within each transect. In the analysed lakes, the C_max_ ranged from 2.0 m to 8.5 m and the isobath next to the C_max_ for a lake was taken for the calculation of the deep littoral slope (Sl_Cmax_). This factor was used as an environmental variable for the submerged and floating-leaved (so-called true hydrophyte) vegetation. Within the 260 analysed transects, the Sl_Cmax_ ranged between 0.12° and 15.93° (median Me = 3.74, standard deviation σ = 3.653) or a slope of 0.2 to 28.5%. For the communities of rush and wetland vegetation (helophytes), a slope in the shallow littoral zone to a depth of 1 m (Sl_1m_) was calculated. The values of Sl_1m_ ranged from 0.05° to 14.23° (Me = 3.34, σ = 2.710) or a slope of 0.1 to 25.4%.

### 2.4 Wind exposure calculation

For each transect the wind exposure was determined according to the approach by Brodersen (1995). First, the effective fetch of a site (transect) was determined by measuring the length of the potential uninterrupted wind route for five lines, the first one connecting perpendicularly to a transect starting point and the opposite lake shore and the other four lines plotted at each side of the first line at the angles of 22.5° and 45.0°. Ultimately, the angle between the extreme lines was 90°. The weighted average of the length of those lines was calculated, with the cosine of the angle with the middle line used as a weight, 1.0 for the middle line, 0.92388 for lines forming a 22.5° angle and 0.70711 for lines forming a 45.0° angle with the middle line (see Bielczyńska, 2019 for details). For the 260 analysed transects, the mean effective fetch ranged between 0.07 and 2.57 km (Me = 0.59, σ = 0.474).

In addition to effective fetch, the wind exposure calculation employed weather conditions, i.e., wind direction and speed during the defined period prior to biological surveys (Brodersen, 1995), combined in the following formula:


$$\text{E}=log\left(1+\text{f}\times \text{w}\times \text{h}\times {\text{d}}^{-2}\right)$$

where: *E* - wind exposure (unitless), *f* – mean effective fetch (km), *w* – share of days in selected period with wind directed towards the site, *h* – mean wind speed (m*s^-1^), *d* – sites depth (m).

Meteorological data were taken from seven meteorological synoptic stations (Institute of Meteorology and Water Management–National Research Institute; public access at: https://danepubliczne.imgw.pl) located nearest to the investigated lakes (**Figure 1**). The distance between the investigated lakes and the meteorological stations ranged between 4 and 54 km, which is considered a distance representative for meteorological data (<100 km; WMO, 2018). The hourly measurements of the direction and the speed of wind within days for the 6-year period preceding macrophyte sampling were averaged with consideration of separate principles of averaging directions (Mardia and Jupp, 2000), according to the following formula:


$$\text{LDM}=\text{arctan}\frac{\sum_{\text{i}=1}^{\text{n}}\text{sin}\theta_{\text{i}}}{\sum_{\text{i}=1}^{\text{n}}\text{cos}\theta_{\text{i}}}$$

where: LDM – *Linear Directional Mean*; *Ѳ_i_* – directions from individual measurements, with correction for the quadrant according to the following algorithm:

- if $\sum_{i=1}^{n}\sin\theta_{i}\geq 0$ and $\sum_{i=1}^{n}\cos\theta_{i}>0\;$ the value of |LDM| is used;
- if $\sum_{i=1}^{n}\sin\theta_{i}\geq 0$ and $\sum_{i=1}^{n}\cos\theta_{i}<0\;$ the value of 180 –|LDM| is used;
- if $\sum_{i=1}^{n}\sin\theta_{i}<0$ and $\sum_{i=1}^{n}\cos\theta_{i}>0\;$ the value of 360 – |LDM| is used;
- if $\sum_{i=1}^{n}\sin\theta_{i}<0$ and $\sum_{i=1}^{n}\cos\theta_{i}<0\;$ the value of 180 + |LDM| is used.

The site depth (*d*) was assumed as 1 m for the analyses of helophytes (rush vegetation) and the maximum colonisation depth (Cmax) of a lake for the analyses of hydrophyte communities (submerged vegetation and those with floating leaves). For calculations, two custom made models in ArcGIS Pro 2.3 (ESRI, 2019) were designed using the Model Builder program and scripts in Python (Bielczyńska, 2019). For the 260 analysed transects, the wind exposure for a shallow littoral zone (Exp_1m_) ranged from 0.015 to 0.669 (Me = 0.15, σ = 0.103), and for the deep littoral zone (Exp_Cmax_) from 0.0007 to 0.356 (Me = 0.007, σ = 0.049).

### 2.5 Statistical analyses

The relationships between environmental variables (wind exposure, littoral slope, TP) and macrophyte indices (as listed in **Supplementary Table 2**) as well as the syntaxonomic composition of aquatic and rush vegetation (as listed in **Supplementary Table 3**) were analysed. First, the effects of explanatory variables on plant community composition were analysed using multidimensional ordination techniques (canonical correspondence analyses or redundancy analysis depending on the biological gradient length), for hydrophye and helophyte vegetation separately. Variation partitioning (VP) was applied to reveal the relative contribution of each environmental variable in explaining hydrophyte and helophyte variation. Second, similarity percentage analysis (SIMPER) was conducted to identify syntaxa associated with littoral slopes of different steepnesses. Third, correlation and regression analyses were used to explore links between environmental variables and macrophyte compositional and abundance metrics. The analyses were performed at the transect level and, additionally, at the lake level (effects on overall lake assessment).

#### 2.5.1 Transect-level analyses

We used multidimensional ordination techniques to explore the effect of environmental variables on macrophyte syntaxonomic composition. Macrophyte status assessment in Poland includes both hydrophyte and rush vegetation. Therefore, both groups were included in this study, though analysed separately. The relative abundance of syntaxa with more than three observations were used as response variables that reduced the number of hydrophyte communities to 39, and helophyte communities to 19. The length of the gradients in biological data (*β*-diversity) were checked using detrended correspondence analysis (DCA). The gradient length along the first canonical axis suggested unimodal data distributions for both hydrophytes and helophytes (SD = 4.21 and 3.32, respectively), hence, canonical correspondence analysis (CCA) was further applied to transect data (ter Braak and Šmilauer, 2002). For helophytes, environmental variables included littoral slope (Sl_1m_) and wind exposure of the shallow littoral zone 0–1 m (Exp_1m_), and for hydrophytes, the littoral slope (Sl_Cmax_) and the wind exposure for deep littoral zone up to a maximum colonisation depth (Exp_Cmax_). Due to a clear variation in trophic conditions (**Supplementary Table 1**), the ln-transformed values of the total phosphorus (lnTP) were additionally used as a proxy of water trophy. Total phosphorus was not correlated with any of the physical variables tested (all Spearman’s correlations non-significant at p>0.05). To determine the relative importance of tested environmental factors in explaining the variation in biological data, the forward selection was used. The statistical significance of each variable was tested using a Monte-Carlo permutation test with 1,000 permutations.

To examine the relative effects of littoral slope, wind exposure and total phosphorus on the macrophyte syntaxonomic composition on the transect-level, VP was applied. This analysis was intended to reveal both the pure and shared effects of explanatory variables (here Sl, Exp and lnTP) on the response variables (here the relative abundance of hydrophytes and helophytes, separately). Variation partitioning was performed using the *varpart* function in the R package *vegan* (Oksanen et al., 2022), for hydrophyte and helophyte communities, separately.

To identify plant communities that most contributed to the differences between transects of different slopes, we performed SIMPER using PAST software (Hammer et al., 2001). Based on the literature data and Sl_Cmax_, transects were divided into three groups of slope steepness: gentle sloped<1.32° or<2.3% (Duarte and Kalff, 1986; van Nes et al., 2002; He et al., 2019), steep sloped >8.42° or >14.8% (Duarte and Kalff, 1990) and intermediate. Communities discriminating for transects with gentle and steep slopes were identified based on the indicator value (IndVal), which relates the relative abundance to the relative frequency of occurrence of the taxa in each group. The IndVal values range between 0 and 1. Communities with IndVal ≥0.50 and significance p<0.05 were considered indicators (Dufrene and Legendre, 1997; Cáceres and Legendre, 2009). We calculated IndVal using the *labdsv* package (Roberts, 2023) in software R (4.3.1 version; R Core Team, 2023) in the RStudio environment (RStudio Team, 2023). As no literature data to distinguish wind exposure groups are available, such analysis was not performed for exposure variables.

To test the effect of environmental variables on macrophyte indices employed in bioassessment, we used correlation analysis between environmental variables (Sl_1m_, Sl_Cmax_, Exp_1m_, Exp_Cmax_) and indicators describing macrophyte composition, diversity and structure on each transect as response variables. The macrophyte indicators included: macrophyte coverage on transects (%COV), absolute number of syntaxa (S_TOT_), number of hydrophyte communities (S_Hy_), number of charophyte communities (S_Ch_), number of helophyte communities (S_He_), relative cover of hydrophyte communities (%N_Hy_), relative cover of charophyte communities (%N_Ch_), diversity index (H’) and the modified assessment index ESMI (ESMI_TR_) (see **Supplementary Table 2** for details). Due to a skewed distribution of all the variables (except for H’) at the transect level (Shapiro-Wilk test p<0.001), the non-parametric Spearman’s rank correlation test was applied. Because the maximum colonisation depth on transects was used to calculate both slope and exposure, this variable was not further used in the analysis. The variation in macrophyte indices on transects with gentle, moderate and steep slopes was compared using the Kruskal-Wallis test with median as a central tendency and the *post-hoc* Mann-Whitney U test.

#### 2.5.2 Lake-level analyses

For lake-level analysis, the values of slope and wind exposure were averaged for each lake, resulting in AvgSl_Cmax_ and AvgExp_Cmax_ for hydrophytes, and AvgSl_1m_ and AvgExp_1m_ for helophytes. Main eutrophication indicators, i.e., total phosphorus (TP), total nitrogen (TN) and Secchi disk visibility (SDV) were also used as response variables at the lake-level, all ln-transformed prior to the analysis. Relative abundance of syntaxa and biological indicators calculated at lake level in accordance with the ESMI method (Ciecierska and Kolada, 2014) were used as response variables (**Supplementary Table 1**). To examine the degree to which variation in each response variable was explained by each of the explanatory variables, we used linear regressions in STATISTICA 10 (StatSoft Inc., 2011). This was reasonable as the lake-level variables approximated normal distribution based on the Shapiro-Wilk test (SW p<0.05).

The relative effects of lake littoral slope, exposure and total phosphorus on macrophyte syntaxonomic composition was explored by variation partitioning using the *varpart* function in the R package *vegan* (Oksanen et al., 2022), for hydrophyte and helophyte communities, separately. The DCA gradient length along the first canonical axis suggested linear data distribution for both hydrophyte and helophyte communities in 16 lakes (SD = 2.60 and 1.19, respectively), therefore, the use of RDA VP was justified (ter Braak and Šmilauer, 2002).

## 3 Results

### 3.1 Effect on syntaxonomic composition at the transect level

#### 3.1.1 Hydrophytes

Three environmental variables (Sl_Cmax_, Exp_Cmax_, lnTP) explained 7.4% variability in hydrophyte syntaxa composition (total inertia = 8.582, sum of all eigenvalues = 0.637). The three canonical axes were statistically significant and they explained 45.4%, 37.8% and 16.8% of the relationship between environmental and dependent variables, respectively. All explanatory variables contributed significantly to the model, with the highest contribution of lnTP (forward selection results: λA = 0.26, F = 7.98, p = 0.002), followed by Exp_Cmax_ (λA = 0.21, F = 6.77, p = 0.002), and Sl_Cmax_ (λA = 0.14, F = 4.61, p = 0.002).

**Figure 2:**
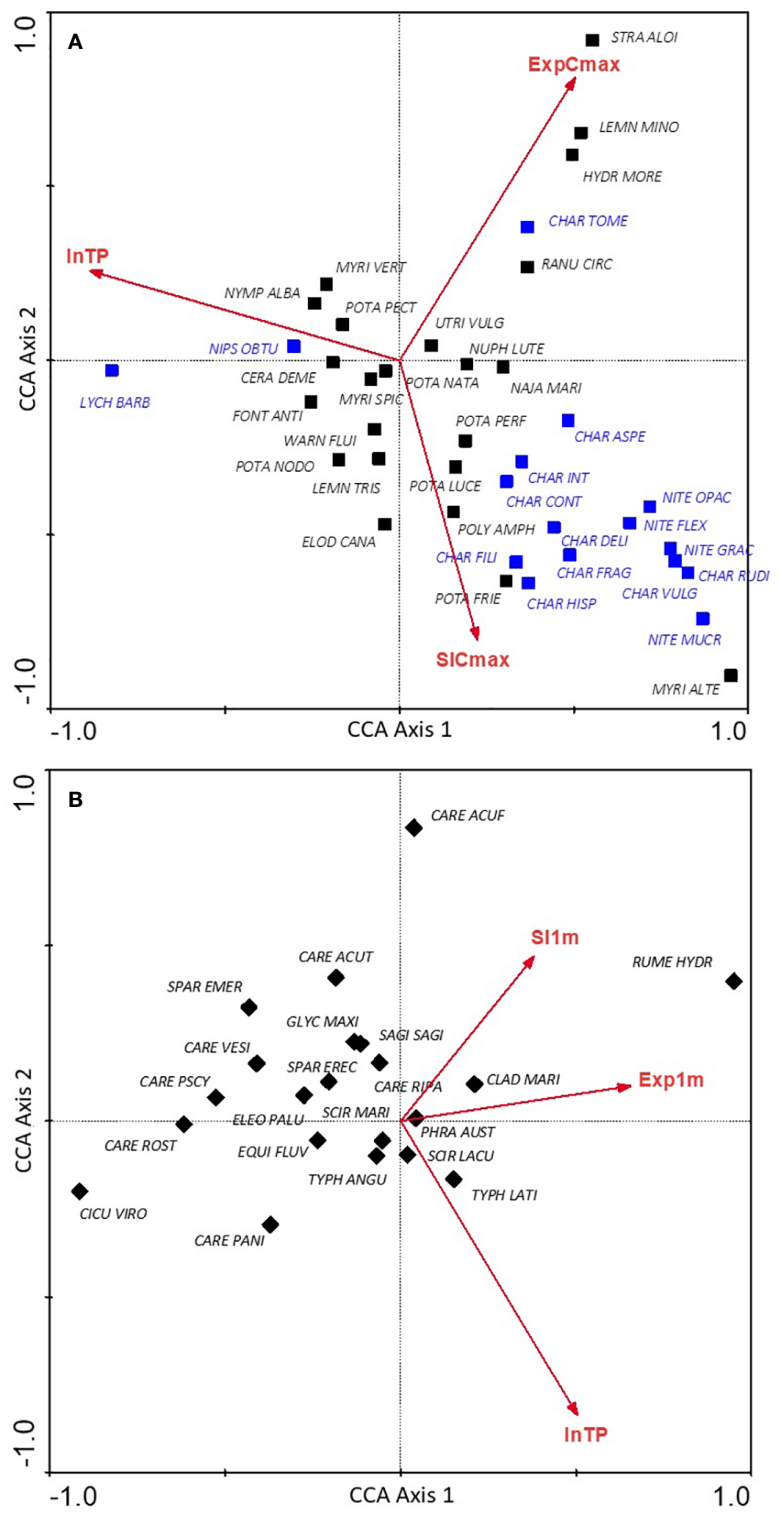
Distribution of macrophyte communities in the canonical ordination space determined by the trophic gradient (lnTP), deep and shallow littoral exposure gradient (Exp_Cmax,_ Exp_1m_) and deep and shallow littoral slope gradient (Sl_Cmax_, Sl_1m_) for hydrophyte **(A)** and helophyte **(B)** communities; explanation of the abbreviations of syntaxa names are provided in **Supplementary Table 3**; charophyte communities are highlighted in blue.

Most noticeably, the vast majority of charophyte communities showed a strong negative association with TP gradient and a positive association with slope gradient (**Figure 2A**). Communities of floating leaved plants, i.e., *Stratiotetum aloidis*, *Lemnetum minoris* and *Hydrocharitetum morsus-ranae* as well as *Charetum tomentosae* and *Ranunculetum circinati* were positively associated with the wind exposure gradient. Communities with the highest frequency in the analysed data pool, except for *Ch. tomentosae*, focused on in the central part of the graph, showing no strong relationships with any of the analysed environmental gradients (indifferent syntaxa, with broad tolerance to habitat physical conditions). The correlations of communities with the three canonical axes are presented in **Supplementary Table 3**.

**Figure 3:**
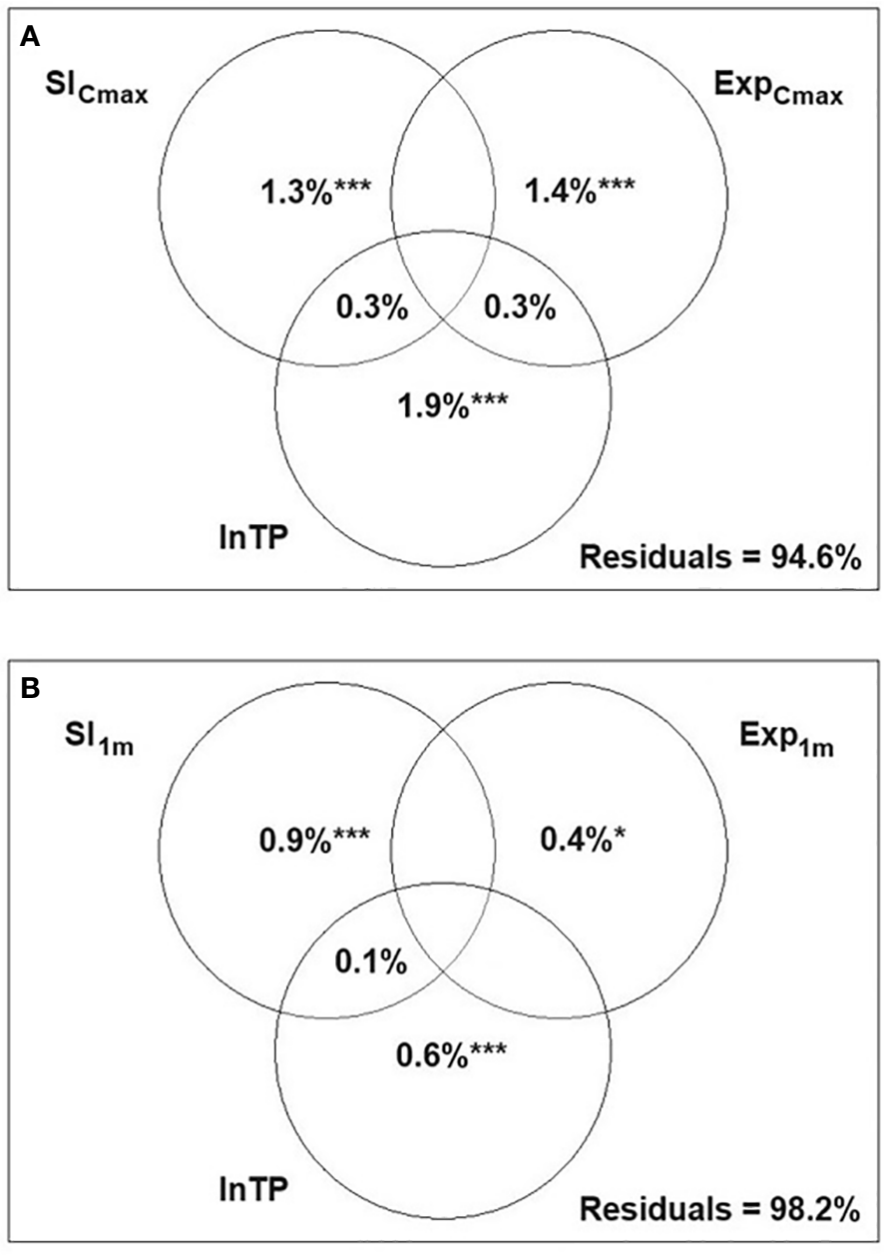
Variance partitioning canonical correspondence analysis (CCA) presenting the relative effects of three variables: littoral slope (Sl_Cmax_, Sl_1m_), wind exposure (Exp_Cmax_, Exp_1m_) and total phosphorus (lnTP) on the syntaxonomic composition of hydrophytes **(A)** and helophytes **(B)** on 260 macrophyte transects; *p<0.05 ***p<0.001; spaces in the diagram with no statistics refer to values<0.

The variation partitioning analysis for hydrophyte communities demonstrated that all three environmental variables contributed significantly to the model, though they explained only a small proportion of the total variance in hydrophyte composition with 89.6% of the variance remaining unexplained. The highest pure effect was found to be for lnTP (**Figure 3A**).

#### 3.1.2 Helophytes

The three environmental variables (Sl_1m_, Exp_1m_, lnTP) explained 3.7% of the variance in helophyte composition data, markedly less than for the hydrophyte communities (total inertia = 4.919, sum of all eigenvalues = 0.181). The three canonical axes were statistically significant and they explained 57.7%, 34.1% and 8.2% of the relationship between environmental and dependent variables, respectively. All explanatory variables contributed significantly to the model, with the highest contribution of lnTP (forward selection results: λA = 0.07, F = 3.83, p = 0.002), followed by Exp_1m_ (λA = 0.06, F = 3.52, p = 0.002), and Sl_1m_ (λA = 0.05, F = 2.36, p = 0.024).

For helophyte communities, links with analysed environmental gradients were weaker than these found for hydrophytes. Most of the communities of emergent vegetation did not show clear relationships with any of the axes (**Figure 2B**), except for the community of great water dock (*Rumex hydrolapathum*), which was clearly associated with transects with higher exposure to the shallow littoral zone, and lesser pond-sedge (*Caricetum acutiformis*) shifted towards slope gradient. Most communities of sedges (*Caricetum* sp.) and ferns (*Thelypteridi-Phragmitetum*) were negatively correlated with the gradient of the littoral slope, while most of the other communities were grouped in the central part of the graph, showing no relationship with the analysed environmental gradients (**Figure 2B**). The values of the correlation of communities with the three canonical axes are included in **Supplementary Table 3**.

For helophyte communities, the pure effects of Sl_1m_ and Exp_1m_ were significant, though they accounted for a small part of the variation (<2.0%), while lnTP appeared insignificant. No shared effect between any of the variables was detected (**Figure 3B**).

#### 3.1.3 Indicator syntaxa

Based on the IndVal analysis, nine communities were determined as indicative of transects with a gentle slope (<1.32°), of which three were formed by floating-leaved plants and four by helophyte species. Of submerged vegetation, *Ranunculetum circinati* and *Charetum tomentosae* appeared indicative of a gentle littoral slope (**Table 1**). The highest IndVal value was attributed to *Statiotetum aloidis*, which showed the strongest association with transects of gentle slopes. With transects of the steep slopes (>8.42°), nine communities of submerged species were associated, with three communities of charophytes and four communities of vascular plants. None helophyte community was indicative of transects with a steep slope. Two communities appeared indicative to moderate slope transects, one community of charophytes *Nitellopsidetum obtusae*, and one of helophytes, *Scirpetum lacustris* (**Table 1**).

**Table 1:** Results of the Indicator Value (IndVal) analysis for the macrophyte communities inhabiting transects with various slope category; charophyte communities marked in blue, helophyte communities marked in bold; for key to macrophyte community full names see **Supplementary Table 3**.

Slope class	Abbreviation	IndVal	p	n
Gentle	*STRA ALOI*	0.621	0.001	58
	*HYDR MORA*	0.419	0.001	23
	***TYPH ANGU***	0.348	0.001	102
	*CHAR TOME*	0.339	0.005	129
	*RANU CIRC*	0.188	0.001	50
	*LEMN MINO*	0.167	0.001	6
	***RUME HYDR***	0.133	0.001	6
	***CICU VIRO***	0.120	0.001	10
	***CARE PSCY***	0.061	0.036	5
Moderate	*NIPS OBTU*	0.406	0.002	188
	***SCIR LACU***	0.267	0.003	65
Steep	*POTA LUCE*	0.309	0.006	98
	*ELOD CANA*	0.242	0.003	30
	*LEMN TRIS*	0.233	0.003	40
	*POTA FRIE*	0.196	0.002	19
	*CHAR FRAG*	0.196	0.008	37
	*CHAR HISP*	0.127	0.002	12
	*CHAR VULG*	0.080	0.012	6
	*POLY AMPH*	0.074	0.038	8
	*MYRI ALTE*	0.061	0.042	5

### 3.2 Effect on abundance and structure indices at the transect level

For macrophyte indices, we found highly statistically significant and moderate to weak relationships between %COV and ESMI_TR_ and the slope of both shallow and deep littoral zones, and most of the macrophyte quantitative indices and wind exposure (**Table 2**). Number and relative abundance of hydrophytes and charophytes were positively correlated with shallow littoral wind exposure, while those of helophytes were negatively correlated.

**Table 2:** Results of the Spearman’s correlation for the macrophyte indicators and the shallow (Sl_1m_) and deep (Sl_Cmax_) littoral slope and shallow (Exp_1m_) and deep littoral wind exposure (Exp_Cmax_) in 260 transects analysed in the study; correlations at p>0.1 not shown, correlations non-significant at p>0.05 are marked with italics.

Macrophyte indices	Abbreviation	Sl_1m_		Sl_Cmax_		Exp_1m_		Exp_Cmax_	
		*R*	*p*	*R*	*p*	*R*	*p*	*R*	*p*
Macrophyte coverage	%COV	-0.19	0.002	-0.25	<0.001				
Total number of communities	S_TOT_							*0.10*	*0.092*
Number of helophyte communities	S_He_					-0.13	0.034		
Number of hydrophyte communities	S_Hy_					0.14	0.024	0.14	0.025
Number of charophyte communities	S_Ch_					0.15	0.018		
Relative cover of hydrophyte communities	%N_Hy_	*-0.11*	*0.095*			0.14	0.020		
Relative cover of charophyte communities	%N_Ch_	*-0.12*	*0.056*			0.15	0.019		
Phytocenotic diversity index	H’					0.15	0.019	0.16	0.008
Ecological State Macrophyte Index for transect	ESMI_TR_	0.26	<0.001	0.33	0.001	0.16	0.009		

**Figure 4:**
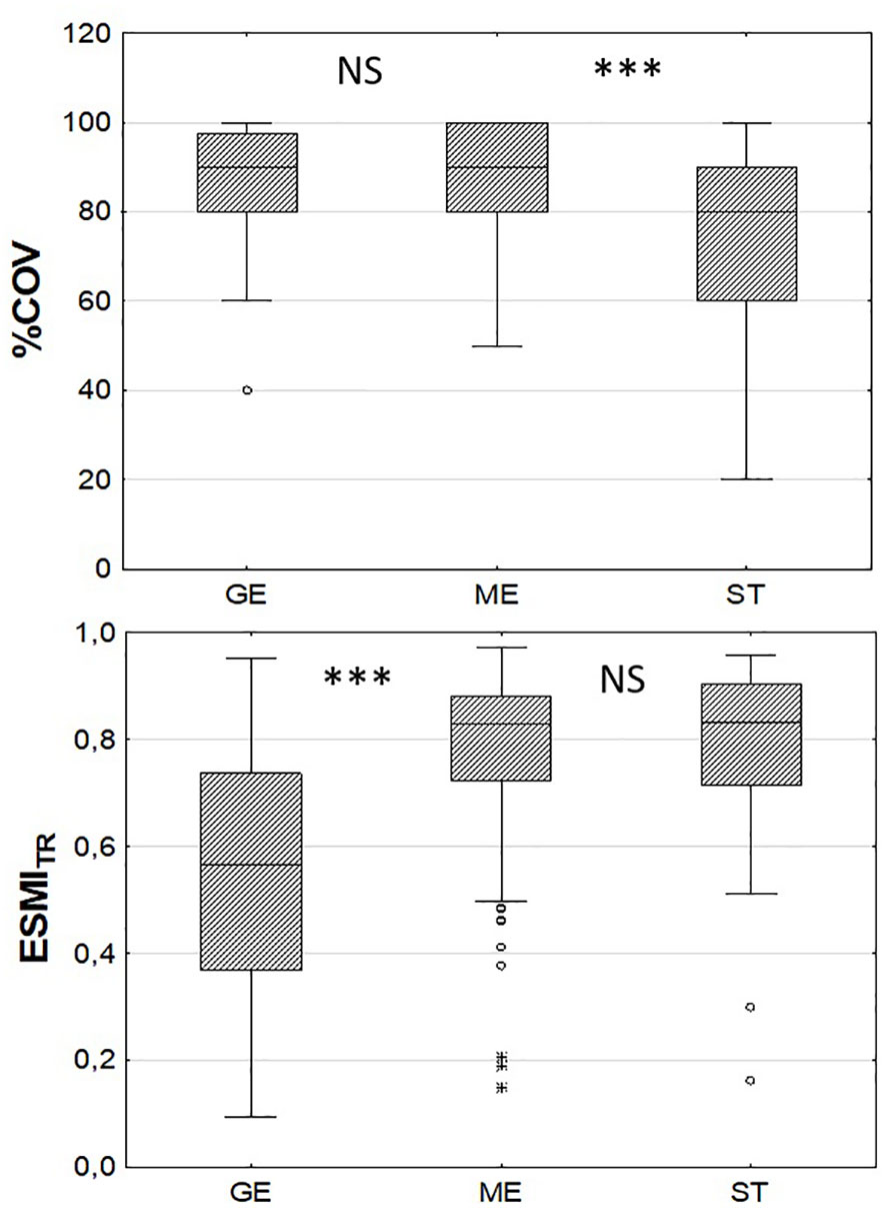
Comparison of central tendencies of macrophyte percentage coverage on transects (%COV) and Ecological Status Macrophyte Index for transects (ESMI_TR_) in three groups of transects with different littoral steepness: GE - gentle (<1.32 °, n=36), ME – moderate (n=180), ST – steep (>8.42°, n=44); ***p<0.001, NS – statistically non-significant based on the *post-hoc* Mann-Whitney U test.

Comparison of the variation in macrophyte indices across groups of transects with gentle (<1.32°, n=36), moderate (n=180) and steep (>8.42°, n=44) slopes showed significant differences only for %COV (K-W: H (2, 260) = 25.07, p< 0.0001) and ESMI_TR_ (K-W: H (2, 258) = 37.28, p< 0.0001). The *post-hoc* Mann-Whitney U test showed statistically significant lower values of ESMI_TR_ on transects with a gentle slope and lower coverage on transects with steep slopes (**Figure 4**).

### 3.3 Effect on lake-level assessment results

For macrophyte indicators analysed at the lake-level, most of them showed no statistically significant relationships with the tested physical factors. The only significant relationships with the littoral slope were found for ESMI and S_Ch_ (**Table 3**) with steeper slopes promoting higher number of charophyte communities and higher ESMI values indicating better ecological status. The slope of both shallow and deep littoral zones strongly correlated with the total nitrogen and water transparency. No effects of physical factors on total phosphorus were detected.

**Table 3:** Linear regression results for macrophyte indicators used in the assessment of the ecological condition of lakes and the average littoral slopes (AvgSl_1m_, AvgSl_Cmax_) and wind exposures (AvgExp_1m_, AvgExp_Cmax_) in 16 lakes analysed in the study; correlations with p>0.1 not shown; correlations non-significant at p>0.05 are marked with italics; for key to macrophyte indices see **Table 2**.

Variable group	Variables	AvgSl_1m_			AvgSl_Cmax_			AvgExp_1m_			AvgExp_Cmax_		
		*R*	*R^2^*	*p*	*R*	*R^2^*	*p*	*R*	*R^2^*	*p*	*R*	*R^2^*	*p*
Trophy	LnTP												
	LnTN	0.71	0.51	0.002	0.84	0.70	<0.001				0.52	0.27	0.038
	LnSD	0.59	0.35	0.015	0.66	0.43	0.006						
Macrophytes	%COV							0.55	0.30	0.035			
	S_TOT_				*0.48*	*0.23*	*0.059*						
	S_He_				*0.47*	*0.20*	*0.083*						
	S_Hy_												
	S_Ch_	0.49	0.24	0.046	0.56	0.31	0.025						
	%N_Hy_												
	%N_Ch_												
	H’												
	ESMI	0.59	0.34	0.017	0.58	0.33	0.020						

**Figure 5:**
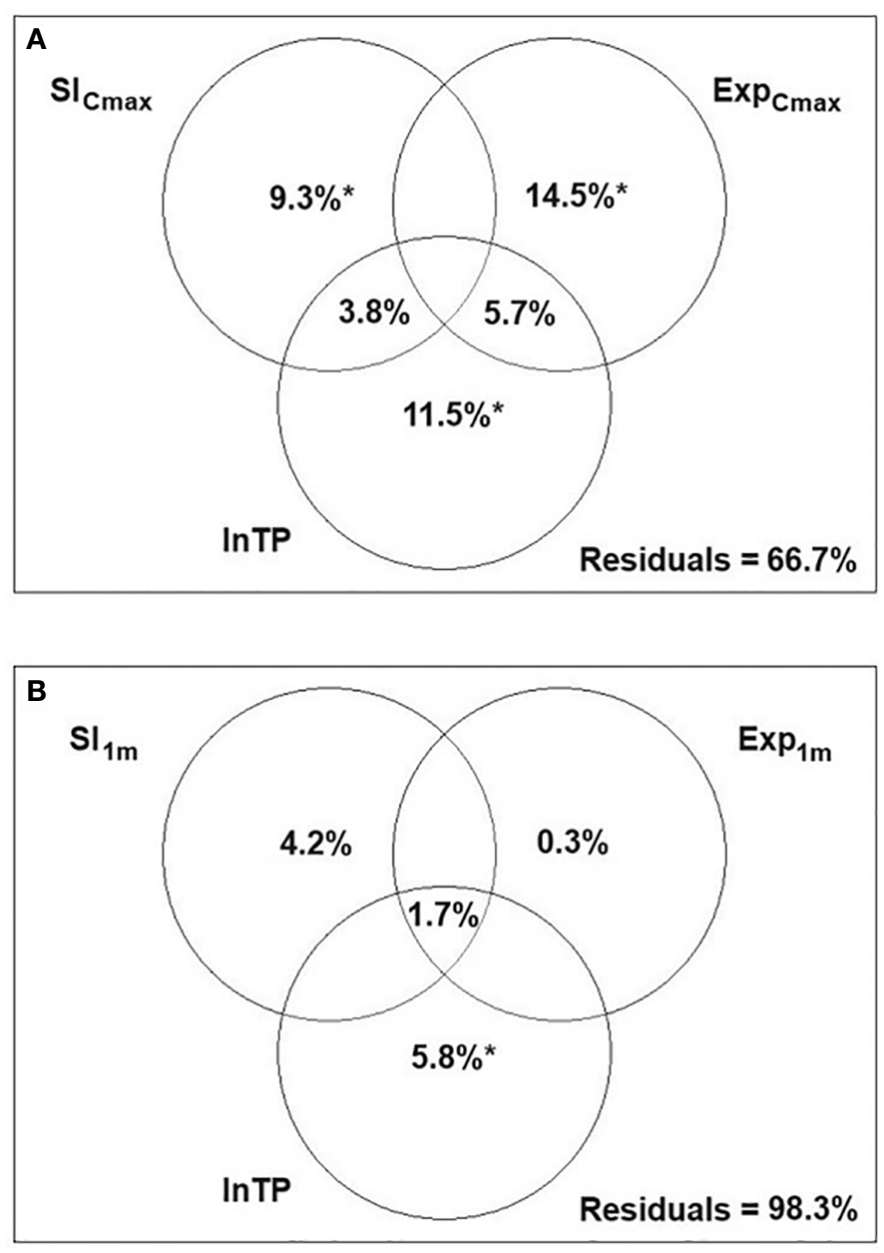
Variance partitioning redundancy analysis (RDA) presenting the relative effects of three variables: littoral slope (Sl_Cmax_, Sl_1m_), wind exposure (Exp_Cmax_, Exp_1m_) and total phosphorus (lnTP) on the syntaxonomic composition of hydrophytes **(A)** and helophytes **(B)** in 16 Polish lowland lakes; *p<0.05; spaces in the diagram with no statistics refer to values<0.

The variation partitioning RDA for hydrophyte communities in 16 lakes revealed that all three variables contributed significantly to the model, with the highest pure effect of wind exposure. The pure effects of all three variables, however, contributed similarly (**Figure 5A**). For helophytes, the unexplained variance was as high as 98.3%. The highest and significant pure effect was attributed to lnTP, while those of slope and exposure of shallow littoral zone were low, negligible and insignificant (**Figure 5B**).

## 4 Discussion

### 4.1 Slope and exposure had minor effect on macrophytes in Polish lakes

Of the three tested environmental variables (littoral slope, wind exposure and water trophy), in almost all cases the latter had the strongest impact on the hydrophyte assemblages, while the effect of the first two was at most moderate, weak or statistically insignificant. These dependencies can be explained by the small values of exposure and slopes found in Polish lakes in general and by the superior role of water trophy in shaping biological assemblages of lakes, mainly autotrophs, phytoplankton and macrophytes (Lyche Solheim et al., 2013). The values of slope and wind exposure observed in the analysed lakes were relatively low compared with those reported in studies from other countries. Statistically significant effects of physical factors on macrophyte characteristics were demonstrated in lakes larger than those in Poland, with an area of tens (Bertrin et al., 2018; Ribaudo et al., 2021) or even hundreds of square kilometres (Riis and Hawes, 2003; Mason et al., 2018; He et al., 2019). Most such studies focus on shallow or very shallow lakes, as the effect of water movements is strongest in subsurface layers and decreases with depth due to absorption of the wave energy by waters. However, the lakes analysed in this study represented morphometric conditions typical of Poland in terms of area and depth as 90% of lakes in the country do not exceed 5 km^2^ (2.9% >10 km^2^) and 98% reach an average depth of 15 m (Kolada et al., 2005).

Although our study covered non-impacted ecosystems, we found that in lakes with steeper littoral slopes, higher water transparency and lower nitrogen concentrations occurred (**Table 3**). These relationships resulted from the well-established links between trophic status and lake morphometry (Vollenweider, 1974; Duarte and Kalff, 1990; Håkanson, 2005), where deeper lakes with more water have a greater capacity to effectively dissolve substances than shallow lakes with less water. Moreover, slope and wind exposure are directly related to lake area and depth, and the latter is known to shape lake vegetation, with deeper and larger lakes promoting higher taxonomic richness and maximum colonisation depth (Søndergaard et al., 2013; Azzella et al., 2014; Kolada, 2014; He et al., 2019). Furthermore, Azzella et al. (2014) demonstrated that at similar water transparency (the same Secchi disk reading values), macrophytes would colonise littoral zones deeper in deep lakes than in shallow ones. Authors attributed this to temperature conditions and thermocline depth. Although some studies indicated temperature as an important factor determining the spatial arrangement of submerged plants (Barko et al., 1986; Dale, 1986; Bornette and Puijalon, 2011), this matter is still insufficiently explored.

### 4.2 Do slope and exposure matter for submerged vegetation?

Our study revealed a clear variation in submerged macrophyte communities concerning preferences towards littoral slope but not wind exposure. It should be stressed, however, that the list of communities analysed in our study was significantly reduced due to the use of the least degraded ecosystems and it did not include syntaxa typically associated with more eutrophied waters. We found, unexpectedly, that charophyte communities were positively associated with littoral slope. Littoral slope is directly related to lake depth, which in turn show strong negative correlation with eutrophication rate (Vollenweider, 1974; Liu et al., 2010; Qin et al., 2020). A wide spectrum of photosynthetic pigments (Schagerl and Pichler, 2000) and high adaptation to shading (Pełechaty, 2006) enable charophytes to colonise the deepest parts of the littoral zone, commonly inaccessible to other plant species (Schwarz et al., 2000; Pełechaty, 2006). The other advantage of charophytes that promotes their colonisation at steeper slopes is that they do not root but anchor with rhizoids; hence, instability of sediments does not hamper their establishment as it does for higher plants. In areas with increased water fertility, higher plants are competitively stronger than charophytes (Blindow et al., 2014). Thus, charophytes may favour deeper lakes with steeper slopes, as these lakes are usually less fertile than shallow ones. On the other hand, shallow littoral zones, where light and nutrients are commonly unlimited, creates an attractive habitat for many species that compete primarily for space. Among charophyte communities, a clear variation in the ability to colonise different littoral slopes was demonstrated (**Table 1**). While communities of *Ch. fragilis*, *Ch. hispida* and *Ch. vulgaris* tended to inhabit transects with steeper slopes, these of *Ch. tomentosae* occurred more frequently and abundantly on transects with gentle slopes typical of shallow littoral zones.

Water fertility, however, is only one of an array of factors that determine the habitat niche occupied by a species. The communities of *Nitellopsis obtusae* and *Chara tomentosa* were shown to occupy similar habitats concerning trophic conditions (Kolada, 2021), whereas in this study we found they are indicative of different littoral slopes. The preferences of submerged plants to occupy different parts of the littoral zones results also from their tolerance to mechanical stress caused by water movements, hence, is determined, to a high degree, by growth-form (Puijalon et al., 2011; Bertrin et al., 2018; He et al., 2019). Low and compact rosette-type species exhibit higher tolerance to water movements than canopy-forming species with tall and slender stems. Likewise, the alternative response strategies to low light conditions of the species with these two growth forms affects their ability to inhabit different basin slopes and water depths. Thanks to their elongated photosynthetic organs, which compensate for light deficits at greater depths, canopy-forming species are able to occupy deeper, steeper parts of the littoral zone (i.e., communities of *Potamogeton lucens* or *Elodea canadensis* in this study). In contrast, the rosette-type hydrophyte species tend to inhabit shallow parts (i.e., communities of *Stratiotes aloidis* in this study, **Table 1**).

### 4.3 Do slope and exposure matter for emergent vegetation?

Different as it is for hydrophytes, which are strongly dependent on water nutrients and light availability, emergent vegetation is shaped mainly by the bottom substrate and sediment hydration (Wetzel, 1988; Barko and James, 1998; Johnston and Brown, 2013; Angiolini et al., 2019). Most of the helophyte communities in our study showed no clear relationship (positive or negative) with the analysed environmental factors, except for the group of marsh species, sedges (*Carex* spp.) and ferns (*Thelypteridis palustris* Schott.), which were negatively correlated with littoral slope gradient (**Figure 2B**). Those species preferred marshy habitats, developing on flat, highly hydrated shores.

In addition to type and hydration of the bottom substrate, other studies also emphasised altitude and alkalinity as important factors determining plant distribution patterns (Kolada et al., 2014; Alahuhta et al., 2017; Angiolini et al., 2019). Our study addresses, however, highly alkaline lakes typical of Polish lowlands, which limited our ability to test these two factors.

As indicated before, the list of communities analysed in our study was limited mainly to those identified in least degraded and eutrophied ecosystems. Therefore, in our study, nitrophilic communities such as *Acoretum calami*, *Phalaridetum arundinaceae*, *Typhetum latifoliae*, *Glycerietum maximae* or *Sparganietum erecti* appeared with a very low frequency (**Supplementary Table 3**), though they are common to Polish lakes (Kolada, 2016). The dominant species of these communities, such as *Typha latifolia* L., *Glyceria maxima* (Hartm.) Holmb. or *Acorus calamus* L., show increased abundance mainly in fertile habitats and are usually not recorded in nutrient-poor habitats (Dykyjová, 1980; Čížková et al., 2001).

### 4.4 Do wind exposure and slope modify assessment results?

In our study, ESMI values at both transect (**Table 2**) and lake level (**Table 3**) positively responded to littoral slopes in both littoral depth zones and wind exposure in the shallow littoral zone. This indicates that transects with a steeper slope and stronger wind exposure had a statistically better ecological status than those with a gentle slope that were sheltered from the wind. This observation was unexpected. The biomass of submerged vegetation was shown to decrease rapidly at a slope greater than 2.24% (Duarte and Kalff, 1986) or 2% (van Nes et al., 2002). Duarte and Kalff (1990) also reported the lack of vegetation at slopes exceeding 14.8% in 25 glacial lakes of North America. On the other hand, He et al. (2019) in Lake Erhai found no loss of vegetation at slopes above 6%, while the most rapid decrease in macrophyte biomass was observed in slopes ranging from 0 to 2%. In the pool of transects analysed in this study, 46 had a slope exceeding 8.42° and most of them were densely vegetated to the maximum colonisation depth (**Figure 4**). Lower values of ESMI, indicating a worse ecological condition on transects with a gentle slope, found in our study may have resulted from the smaller colonisation depth observed there, and also from the faster rate of eutrophication of shallow lakes compared to deep lakes with greater slopes.

Negative relationships between the macrophyte index ESMI and the water trophic status were obvious, as this index was developed and calibrated for assessing eutrophication (Ciecierska and Kolada, 2014; Kolada et al., 2020). The links between the index and littoral slope may, however, have resulted from the relationships between lake morphometry and water trophy (Vollenweider, 1974; Liu et al., 2010; Qin et al., 2020). As steep slopes are usually associated with increased depth, lakes with steep littoral zones tend to maintain better water quality than shallow lakes and support favourable conditions for the development of charophyte communities. Charophytes are associated with waters of good quality and their abundant occurrence is indicative of good ecological status (Kolada, 2021). Therefore, lakes with a higher proportion of steep-sloped transects may potentially have a higher abundance of charophyte communities and a statistically better ecological status than shallow lakes with gentle slopes. This was confirmed by statistically lower ESMI_TR_ values found in the group of transects with gentle slopes <2° (**Figure 4**).

## 5 Conclusions

Contrary to our hypothesis, we found no negative effects of littoral slope and wind exposure on macrophyte growth and development in the analysed lakes. Links between the ecological status and slope and wind exposure, even if statistically significant, were relatively weak.

Unexpectedly, our study demonstrated that littoral slope influenced taxonomic composition of aquatic vegetation, although this effect was opposite to what we expected as steep slopes promoted the diversity of charophytes. The ability of stoneworts to colonise deep and steep littoral slopes most likely results from their adaptation to shading and sediment instability, which favours their establishment over higher plants. This relationship resulted in a higher classification of transects located in steeper parts of the littoral zone than those of gentle slopes.

Although we focused our research on unimpacted lakes where eutrophication-derived modifications did not mask macrophyte characteristics inherent to abiotic natural conditions, the dominant factor explaining variability in macrophyte communities was water fertility. Apparently, in lowland lakes with relatively small areas and low wind exposure typical of European plains, eutrophication is a much more important factor determining aquatic vegetation and the macrophyte-based ecological status than physical features.

Because littoral slopes and wind exposures in ranges typical of lowland lakes in Poland appeared unobtrusive and did not negatively affect lake classification based on macrophytes, we found no basis for recommending modifications to the current national classification system towards mitigating the effects of the analysed physical factors.

## Data availability statement

The original contributions presented in the study are included in the article/**Supplementary Material**. Further inquiries can be directed to the corresponding author.

## Author contributions

AK: Conceptualization, Formal analysis, Funding acquisition, Methodology, Writing – original draft, Visualization, Writing – review & editing. SK: Formal analysis, Methodology, Conceptualization, Visualization, Writing – review & editing. AB: Methodology, Writing – review & editing.
