# Supplementary material for: Minor effect of wind exposure and littoral slope on macrophyte characteristics in non-impacted lowland lakes of Poland
Source: Front Plant Sci. 2024 Jan 9;14:1307453. doi: 10.3389/fpls.2023.1307453 (PMC10803623; doi:10.3389/fpls.2023.1307453)
Supplement: Supplementary file 1 [file DataSheet_1.pdf]

Supplementary Material

Table S1. Basic morphometric, hydrochemical and botanical characteristics of lakes analysed in the study (n = 16); Me - median, Min-max – range, σ - standard deviation

Features	Parameters [abbreviation]	unit	Me	Min-Max	σ
Morphometric	Area [A]	km^2	1.52	0.53-6.80	1.718
	Maximum depth [Z_{max}]	m	22.0	2.5-43.0	13.91
	Mean depth [Z_{mean}]	m	7.2	0.6-14.7	3.95
	Perimeter [P]	km	9.8	4.4-18.9	4.45
Physicochemical	pH		8.3	7.9-8.4	0.15
	Alkalinity	meq l^{-1}	2.5	1.2-3.5	0.60
	Conductivity	µS cm^{-1}	288	177-416	70.2
	Total phosphorus [TP]	mgP l^{-1}	0.027	0.015-0.049	0.0107
	Total nitrogen [TN]	mgN l^{-1}	0.93	0.42-1.95	0.383
	Secchi disk reading [SD]	m	4.0	1.6-6.4	1.31
Macrophytes	Maximum colonisation depth [C_{max}]	m	4.8	1.5-6.4	1.44
	Macrophyte coverage [%COV]	%	86.2	74.04-100	6.85
	Total number of communities [S_{TOT}]	-	24	13-40	7.91
	Number of helophyte communities [S_{He}]	-	8	3-14	3.4
	Number of hydrophyte communities [S_{Hy}]	-	17	10-32	5.6
	Number of charophyte communities [S_{Ch}]	-	4	2-16	3.4
	Relative cover of helophyte communities [%N_{He}]	%	24.7	12.6-36.1	9.15
	Relative cover of hydrophyte communities [%N_{Hy}]	%	75.3	63.1-87.4	9.00
	Relative cover of charophyte communities [%N_{Ch}]	%	57.8	13.5-97.8	23.96
	Phytocenotic diversity index [H’]	-	2.1	1.2-3.0	0.48
	Ecological State Macrophyte Index [ESMI]	-	0.71	0.48-0.79	0.084

Table S2: Macrophyte composition, abundance and structure metrics analysed for transects (n = 260); Me - median, Min-max – range, σ - standard deviation

**Macrophyte indices**	**Me**	**Min-Max**	**σ**
C_{max}	5	0.5-8.5	1.74
%COV	90	20-100	14.7
S_{TOT}	8	3-20	3.3
S_{He}	2	1-10	1.4
S_{Hy}	6	1-15	2.7
S_{Ch}	2	0-10	1.7
$\%N_{Hy}$	81.3	0.7-99.4	19.65
$\%N_{Ch}$	42.4	0-97.4	27.83
H’	1.38	0.07-2.44	0.440
$ESMI_{TR}$	0.809	0.094-0.973	0.1796

Table S3. The list of plant communities analysed in the study and the correlations of communities with the three canonical axes; communities with less than 4 observations were not included in the CCA analysis; n – number of occurrence; F% - frequency of occurrence

Plant community	Abbreviation	n	F%	Axis 1	Axis 2	Axis 3
**HYDROPHYTE SYNTAXA**						
Nitellopsidetum obtusae	NIPS OBTU	189	72,4	-0,296	-0,080	-0,265
Ceratophylletum demersi	CERA DEME	153	58,6	-0,341	-0,079	0,000
Charetum tomentosae	CHAR TOME	129	49,4	0,415	0,772	0,102
Myriophylletum spicati	MYRI SPIC	127	48,7	-0,032	-0,116	0,146
Potametum lucentis	POTA LUCE	99	37,9	0,036	-0,393	0,339
Nymphaeo albae-Nupharetum luteae f. with *Nuphar lutea* (L.) Sibth. & Sm.	NUPH LUTE	79	30,3	-0,315	0,097	-0,028
Najadetum marinae	NAJA MARI	76	29,1	0,611	0,097	0,340
Lemno-Utricularietum vulgaris	UTRI VULG	75	28,7	-0,293	0,132	-0,022
Stratiotetum aloidis	STRA ALOI	58	22,2	0,600	1,720	0,349
Ranuncutetum circinati	RANU CIRC	51	19,5	-0,402	0,637	0,045
Potametum pectinati	POTA PECT	42	16,1	0,085	0,043	-0,665
Fontinaletum antipyreticae	FONT ANTI	41	15,7	-0,381	-0,273	0,323
Lemnetum trisulcae	LEMN TRIS	40	15,3	0,375	-0,501	0,041
Nymphaeo albae-Nupharetum luteae f. with *Nymphaea alba* L.	NYMP ALBA	40	15,3	-0,638	0,149	-0,621
Potametum perfoliati	POTA PERF	40	15,3	0,178	-0,288	-0,037
Charetum fragilis	CHAR FRAG	37	14,2	1,045	-0,708	0,183
Elodeetum canadensis	ELOD CANA	30	11,5	0,338	-0,738	1,004
Warnstorfia fluitans	WARN FLUI	30	11,5	-1,040	-0,242	0,745
Charetum delicatulae	CHAR DELI	28	10,7	0,997	-0,612	-0,012
Lemno-Hydrocharitetum morsus-ranae	HYDR MORA	23	8,8	-0,384	1,214	0,015
Potametum natantis	POTA NATA	22	8,4	-0,159	-0,092	-0,653
Charetum asperae	CHAR ASPE	19	7,3	1,192	-0,127	-0,280
Potametum friesi	POTA FRIE	19	7,3	0,827	-0,866	0,859
Charetum contrariae	CHAR CONT	17	6,5	0,916	-0,478	-0,277
Charetum rudis	CHAR RUDI	16	6,1	1,859	-0,688	-0,690
Charetum filiformis	CHAR FILI	12	4,6	0,939	-0,792	0,490
Charetum hispidae	CHAR HISP	12	4,6	0,860	-0,852	0,789
Nitelletum flexilis	NITE FLEX	11	4,2	1,690	-0,573	-0,795
Nitelletum gracilis	NITE GRAC	11	4,2	1,860	-0,644	-0,807
Charetum intermediae	CHAR INTE	10	3,8	1,262	-0,423	-0,928
Lychnothamnetum barbatii	LYCH BARB	10	3,8	-0,897	-0,343	0,932
Polygonetum natantis	POLY AMPH	8	3,1	-0,557	-0,517	1,163
Charetum vulgaris	CHAR VULG	6	2,3	1,881	-0,714	-0,474
Lemnetum minoris	LEMN MINO	6	2,3	-0,979	1,371	0,126

Plant community	Abbreviation	n	F%	Axis 1	Axis 2	Axis 3
Myriophyllo-Littorelletum f. with *Myriophyllum alterniflorum* DC.	MYRI ALTE	5	1,9	1,880	-1,070	0,538
Myriophylletum verticillati	MYRI VERT	5	1,9	-0,759	0,295	0,002
Nitelletum mucronatae	NITE MUCR	4	1,5	1,868	-0,882	-0,077
Community of *Nitella opaca*	NITE OPAC	4	1,5	1,853	-0,505	-1,257
Community of *Potamogeton nodosus* Poir.	POTA NODO	4	1,5	0,299	-0,551	0,177
Community of *Potamogeton berchtoldii* Fieber	POTA BERC	3	1,1	-	-	-
Charetum tenuispinae	CHAR TENU	2	0,8	-	-	-
Potametum compressi	POTA COMP	2	0,8	-	-	-
Community of *Potamogeton crispus* L.	POTA CRIS	2	0,8	-	-	-
Potametum filiformis f. with *Potamogeton praelongus* Wulfen	POTA PRAE	2	0,8	-	-	-
Community of *Potamogeton trichoides* Cham. & Schecht.	POTA TRICH	1	0,4	-	-	-
Lemno-Spirodeletum polyrrhizae	SPIR POLY	1	0,4	-	-	-
**HELOPHYTE SYNTAXA**						
Phragmitetum communis	PHRA AUST	251	96,2	0,110	0,067	-0,007
Typhetum angustifoliae	TYPH ANGU	102	39,1	-0,007	-0,355	-0,007
Scirpetum lacustris	SCIR LACU	66	25,3	0,212	-0,291	0,270
Caricetum ripariae	CARE RIPA	36	13,8	-0,615	0,598	-0,101
Typhetum latifoliae	TYPH LATI	27	10,3	0,240	0,041	-0,222
Caricetum acutiformis	CARE ACUF	25	9,6	-0,644	0,670	-0,293
Sparganietum erecti	SPAR EREC	20	7,7	-0,590	-0,035	0,372
Caricetum gracilis	CARE ACUT	11	4,2	-1,072	2,240	0,632
Eleocharitetum palustris	ELEO PALU	11	4,2	-0,508	-0,206	-0,326
Cicuto-Caricetum pseudocyperi f. with *Cicuta virosa* L.	CICU VIRO	10	3,8	-0,464	-0,962	0,181
Sagittario-Sparganietum emersi f. with *Sagittaria sagittifolia* L.	SAGI SAGI	7	2,7	-0,829	0,715	-0,359
Equisetetum fluviatilis	EQUI FLUV	6	2,3	-0,968	-0,066	0,128
Zbiorowisko z Rumex hydrolapathum Hudson	RUME HYDR	6	2,3	1,131	0,596	2,647
Cicuto-Caricetum pseudocyperi f. with *Carex pseudocyperus* L.	CARE PSCY	5	1,9	-1,211	-0,462	-0,148
Glycerietum maximae	GLYC MAXI	5	1,9	-0,471	0,340	0,157
Iridetum pseudacori	IRIS PSAC	5	1,9	-0,345	-0,050	-0,160
Thelypteridi-Phragmitetum	THEL PALU	5	1,9	-1,590	-0,086	0,173
Caricetum rostratae	CARE ROST	4	1,5	-1,808	-0,414	0,052
Scirpetum maritimi f.with *Scirpus maritimus* L.	SCIR MARI	4	1,5	-0,544	0,325	-0,707

Plant community	Abbreviation	n	F%	Axis 1	Axis 2	Axis 3
Caricetum vesicariae	CARE VESI	2	0,8	-	-	-
Cladietum marisci	CLAD MARI	2	0,8	-	-	-
Sagittario-Sparganietum emersi f. with *Sparganium emersum* Rehmann	SPAR EMER	2	0,8	-	-	-
Caricetum paniculatae	CARE PANI	1	0,4	-	-	-
